# Supplementary material for: A Pleiotropic Role of Long Non-Coding RNAs in the Modulation of Wnt/β-Catenin and PI3K/Akt/mTOR Signaling Pathways in Esophageal Squamous Cell Carcinoma: Implication in Chemotherapeutic Drug Response
Source: Curr Oncol. 2022 Mar 26;29(4):2326–49. doi: 10.3390/curroncol29040189 (PMC9031703; doi:10.3390/curroncol29040189)
Supplement: Supplementary file 1 [file curroncol-29-00189-s001.zip › curroncol-1604851-supplementary.pdf]

Current Oncology

Supplementary Material

A Pleiotropic Role of Long Non-Coding RNAs in the Modulation of Wnt/β-Catenin and PI3K/Akt/mTOR Signaling Pathways in Esophageal Squamous Cell Carcinoma: Implication in Chemotherapeutic Drug Response

Uttam Sharma [1], Masang Murmu [1], Tushar Singh Barwal [1], Hardeep Singh Tuli [2], Manju Jain [3], Hridayesh Prakash [4], Tea Kaceli [5], Aklank Jain [1,*] and Anupam Bishayee [5,*]

1 Department of Zoology, Central University of Punjab, Ghudda 151 401, Punjab, India
2 Department of Biotechnology, Maharishi Markandeshwar (Deemed to be University), Mullana-Ambala 133 207, Haryana, India
3 Department of Biochemistry, Central University of Punjab, Ghudda 151 401, Punjab, India;
4 Amity Institute of Virology and Immunology, Amity University, Noida 201 301, Uttar Pradesh, India;
5 College of Osteopathic Medicine, Lake Erie College of Osteopathic Medicine, Bradenton, FL 34211, USA
* Correspondence: aklankjain@gmail.com (A.J.); abishayee@lecom.edu, abishayee@gmail.com (A.B)

Table S1. Association of ESCC related lncRNAs in various signalling pathways.

S No.	*LncRNA*	Full Form	Signalling pathways	References
1	*HOTAIR*	HOX Transcript Antisense RNA	Wnt signaling pathway	[67]
2	*LINC RNA-uc003opf.1 or* (*LINC00951*)	Long Intergenic Non-Protein Coding RNA 951	NA	[68]
3	*ANRIL or CDKN2B-AS1*	Cyclin Dependent Kinase Inhibitor 2B Antisense RNA 1	TGFβ1 signaling pathway	[34]
4	*FOXCUT*	Forkhead Box C1 Upstream Transcript	NA	[69]
5	*H19*	H19 Imprinted Maternally Expressed Transcript	IGF2 imprinting pathway	[70]
6	*LINC-POU3F3*	POU Class 3 Homeobox 3	NA	[71]
7	*LOC285194*	Also known as Tumor Suppressor Candidate 7 (*TUSC7*)	NA	[54]
8	*PLncRNA-1 or CBR3-AS1*	CBR3 Antisense RNA 1	NA	[72]
9	*SOX2OT*	SRY-Box Transcription Factor 2 Overlapping Transcript	NA	[73]
10	*SPRY4-IT1*	Sprouty RTK Signaling Antagonist 4 Intronic Transcript 1	NA	[74]
11	*UCA1*	Urothelial Cancer Associated 1	Wnt signaling pathway	[75]
12	*91H*	Long noncoding RNA 91H	NA	[76]
13	*BOKAS or BOK-AS1*	BOK antisense RNA 1	Wnt/β-catenin signaling pathway	[77]
14	*CCAT2*	Colon Cancer Associated Transcript 2	β-catenin/WISP1 signaling pathway	[78]
15	*LINC RNA-uc002yug.2 or LINC01426*	Long Intergenic Non-Protein Coding RNA 1426	NA	[79]
16	*Long noncoding RNA POLR2E*	RNA Polymerase II, I And III Subunit E	NA	[80]
17	*MALAT1*	Metastasis Associated Lung Adenocarcinoma Transcript 1	β-catenin, ATM-CHK2 signaling pathway	[81]
18	*NEAT1*	Nuclear Paraspeckle Assembly Transcript 1	NA	[82]
19	*PCAT-1*	Prostate Cancer Associated Transcript 1	NA	[83]
20	*POU3F3*	POU Class 3 Homeobox 3	NA	[84]
21	*SOX2OT*	SRY-Box Transcription Factor 2 Overlapping Transcript	NA	[85]
22	*TUG1*	Taurine Up-Regulated 1	Wnt/β-catenin, Nrf2 signaling pathways	[86]
23	*ZEB1-AS1*	Zinc Finger E-Box Binding Homeobox 1 Antisense RNA 1	NA	[87]
24	*AFAP1-AS1*	Actin Filament Associated Protein 1 Antisense RNA 1	NA	[88]
25	*BANCR*	BRAF-Activated Non-Protein Coding RNA	IGF1R/Raf/MEK/ERK signaling pathway	[89]
26	*BC032469*		NA	[90]
27	*BC200 or BCYRN1*	Also known as Brain Cytoplasmic RNA 1	NA	[91]
28	*CASC9*	Cancer Susceptibility 9	NA	[92]
29	*HOTTIP*	HOXA Distal Transcript Antisense RNA	NA	[93]
30	*LINC-ROR*	Long Intergenic Non-Protein Coding RNA, Regulator Of Reprogramming	p53 signaling pathway	[94]
31	*LOC100130476 or WAKMAR2*	Wound and Keratinocyte Migration Associated LncRNA 2	NA	[95]

32	*MEG3*	Maternally expressed gene 3	p53 signaling pathway	[96]
33	*SOX2OT and SOX2*	SOX2 Overlapping Transcript and SRY-Box Transcription Factor 2	NA	[97]
34	*ATB*	Long Noncoding RNA Activated By TGF-Beta	NA	[98]
35	*CCAT1*	Colon Cancer Associated Transcript 1	NA	[99]
36	*ESCCAL-1 or CASC9*	Cancer Susceptibility 9	NA	[100]
37	*GHET1*	Gastric Carcinoma Proliferation Enhancing Transcript 1	NA	[101]
38	*HNF1A-AS1*	HNF1 Homeobox A Antisense RNA 1	NA	[102]
39	*HOTAIR*	HOX transcript antisense RNA	NA	[103]
40	*LINC00460*	Long Intergenic Non-Protein Coding RNA 460	NA	[104]
41	*LINCRNA-NR_024015 or TDRG1*	Also known as Testis Development Related 1	NA	[105]
42	*MEG3*	Maternally expressed gene 3	GSK-3β/Snail/ Wnt/β-catenin signaling pathway	[106]
43	*MIR31HG*	MIR31 Host Gene	NA	[107]
44	*NORAD*	Non-Coding RNA Activated By DNA Damage	NA	[108]
45	*Pseudogene PHBP1*	Prohibitin Pseudogene 1	NA	[109]
46	*PVT1*	Pvt1 Oncogene	NA	[110]
47	*RP11-766N7.4 or LINC02231*	Long Intergenic Non-Protein Coding RNA 2231	NA	[111]
48	*SNHG1*	Small Nucleolar RNA Host Gene 1	Notch signaling pathway	[27]
49	*SNHG16*	Small Nucleolar RNA Host Gene 16	Wnt/β-catenin signaling pathway	[112]
50	*XIST*	X Inactive Specific Transcript	NA	[113]
51	*ZFAS1*	ZNFX1 Antisense RNA 1	STAT3 signaling pathway	[114]
52	*AK001796 or MIR4435-2HG*	MIR4435-2 Host Gene	MDM2/ p53 signaling pathway	[115]
53	*DANCR*	Differentiation Antagonizing Non-Protein Coding RNA	NA	[116]
54	*DUXAP10*	Double Homeobox A Pseudogene 10	NA	[117]
55	*DUXAP8*	Double Homeobox A Pseudogene 8	NA	[118]
56	*ECM*	long non-coding RNA-extracellular matrix	NA	[119]
57	*EZR-AS1*	Ezrin Antisense RNA 1	NA	[120]
58	*FAM201A*	Family With Sequence Similarity 201 Member A	NA	[121]
59	*FER1L4*	Fer-1 Like Family Member 4 (Pseudogene)	NA	[122]
60	*FOXD2-AS1*	Forkhead Box D2 Adjacent Opposite Strand RNA 1	Akt signaling pathway	[123]
61	*FTH1P3*	Ferritin Heavy Chain 1 Pseudogene 3	NF-kB signaling pathway	[26]
62	*GAS5*	Growth Arrest Specific 5	NA	[124]
63	*HOXA11-AS*	Homeobox A11 Antisense RNA	NA	[125]
64	*LET*	long noncoding RNA-LET	NA	[126]
65	*LINC00657 or NORAD*	Also known as Non-Coding RNA Activated By DNA Damage	NA	[127]

66	*LINC00675 or* TMEM238L	Also known as Transmembrane Protein 238	Wnt/β-catenin signaling pathway	[13]
67	*LINC01133*	Long Intergenic Non-Protein Coding RNA 1133	NA	[128]
68	*LINC01296 or DUXAP9*	Double Homeobox A Pseudogene 9	NA	[129]
69	*LINC01503*	Long Intergenic Non-Protein Coding RNA 1503	NA	[130]
70	*LINC-UBC1 or UBE2K*	Also known as Ubiquitin Conjugating Enzyme E2 K	NA	[131]
71	*LncRNA CTC-276P9.1 or* EPIST	Esophagus Epithelial Intergenic Associated Transcript	NA	[132]
72	*LUCAT1*	Lung Cancer Associated Transcript 1	NA	[133]
73	*NKILA*	NF-KappaB Interacting LncRNA	NF-κB signaling pathway	[25]
74	*NMR*	NSUN2 methylated lncRNA	ERK1/2 signaling pathway	[134]
75	*PART1*	Prostate Androgen-Regulated Transcript 1	NA	[63]
76	*SBF2-AS1*	SET Binding Factor 2 Antisense RNA 1	NA	[135]
77	*SNHG6*	Small nucleolar host gene 6	NA	[136]
78	*TTN-AS1*	Titin Antisense RNA 1	NA	[137]
79	*TUSC7*	Tumor Suppressor Candidate 7	NA	[55]
80	*UCA1 and MALAT1*	Urothelial Cancer Associated 1 and Metastasis Associated Lung Adenocarcinoma Transcript 1	NA	[138]
81	*CASC2*	Cancer Susceptibility 2	Akt signaling pathway	[139]
82	*DANCR and ZEB1*	Differentiation Antagonizing Non-Protein Coding RNA and Zinc Finger E-Box Binding Homeobox 1	NA	[140]
83	*DLX6-AS1*	Distal-Less Homeobox 6 Antisense RNA 1	NA	[141]
84	*DNM3OS*	DNM3 Opposite Strand/Antisense RNA	NA	[142]
85	*Erbb4-IR*	Erb-B2 Receptor Tyrosine Kinase 4 - IR	NA	[143]
86	*FEZF1-AS1*	FEZ Family Zinc Finger 1 Antisense RNA 1	JAK2/STAT3 signaling pathway, Wnt/β-catenin signaling pathway	[38]
87	*FMR1-AS1*	FMRP Translational Regulator 1 Antisense RNA 1	NFκB/c-Myc signaling pathway	[144]
88	*HAND2-AS1*	Heart And Neural Crest Derivatives Expressed 2 Antisense RNA 1	NA	[145]
89	*HERES*		Wnt signaling pathways	[9]
90	*IRF1-AS*	Interferon Regulatory Factor 1	NA	[146]
91	*LBX2-AS1*	Ladybird Homeobox 2 Antisense RNA 1	NA	[147]
92	*LEF1-AS1*	Lymphoid Enhancer Binding Factor 1 Antisense RNA 1	NA	[148]
93	*LINC00152 or CYTOR*	Cytoskeleton Regulator RNA	NA	[149]
94	*LINC00473*	Long Intergenic Non-Protein Coding RNA 473	NA	[150]
95	*LINC01419*	Long Intergenic Non-Protein Coding RNA 1419	NA	[60]

96	*LINC01518*	Long Intergenic Non-Protein Coding RNA 1518	PIK3CA/Akt signaling pathway	[151]
97	*LINC01980*	Long Intergenic Non-Protein Coding RNA 1980	MYO5A signaling pathway	[152]
98	*LINCRNA-p21 or TP53COR1*	Also known as Tumor Protein P53 signaling Corepressor 1	p53 signaling pathway	[33]
99	*Lnc-ATB*	Long Noncoding RNA Activated By TGF-Beta	NA	[153]
100	*LncRNAXLOC_001659*	Long Noncoding RNA XLOC_001659	NA	[154]
101	*LSINCT5*	Long Stress-Induced Non-Coding Transcript 5	NA	[155]
102	*MNX1-AS1*	Motor Neuron And Pancreas Homeobox 1 Antisense RNA 1	NA	[156]
103	*NR2F1-AS1*	Nuclear Receptor Subfamily 2 Group F Member 1 Antisense RNA 1	Hedgehog signaling pathway	[32]
104	*PANDA or* PANDAR	Also known as Promoter Of CDKN1A Antisense DNA Damage Activated RNA	NA	[157]
105	*PCAT1*	Prostate Cancer Associated Transcript 1	NA	[158]
106	*PGM5-AS1*	Phosphoglucomutase 5 Antisense RNA 1	NA	[159]
107	*SEMA3B-AS1*	Semaphorin 3B Antisense RNA 1	NA	[160]
108	*SNHG20*	Small Nucleolar RNA Host Gene 20	ATM-JAK-PD-L1 signaling pathway	[161]
109	*SNHG6*	Small Nucleolar RNA Host Gene 6	NA	[162]
110	*SNHG8*	Small Nucleolar RNA Host Gene 8	NA	[163]
111	*SPINT1-AS1*	Serine Peptidase Inhibitor, Kunitz Type 1 Antisense RNA 1	NA	[164]
112	*TP73-AS1*	Tumor Protein P73 Antisense RNA 1	NA	[165]
113	*ZNF667-AS1*	Zinc Finger Protein 667 Antisense RNA 1	NA	[166]
114	*AGPG or ACTG1P25*	Actin Gamma 1 Pseudogene 25	NA	[167]
115	*CASC8*	Cancer Susceptibility 8	NA	[168]
116	*EGFR-AS1*	Epidermal Growth Factor Receptor Antisense RNA 1	NA	[169]
117	*FAM225A*	Family With Sequence Similarity 225 Member A	NA	[170]
118	*FAM83H-AS1 or IQANK1*	IQ Motif And Ankyrin Repeat Containing 1	NA	[171]
119	*FGD5-AS1*	FYVE, RhoGEF And PH Domain Containing 5 Antisense RNA 1	NA	[172]
120	*FOXP4-AS1*	Forkhead Box P4 Antisense RNA 1	NA	[173]
121	*HCG22*	HLA Complex Group 22	NA	[174]
122	*HEIH*	Hepatocellular Carcinoma Up-Regulated EZH2-Associated Long Non-Coding RNA	NA	[175]
123	*IUR*	Imatinib-up-regulated lncRNA	NA	[176]
124	*KLF3-AS1*	KLF3 Antisense RNA 1	NA	[177]

125	*LINC00278*	Long Intergenic Non-Protein Coding RNA 278	AR Signaling pathway	[178]
126	*LINC00337*	Long Intergenic Non-Protein Coding RNA 337	NA	[51]
127	*LINC00662*	Long Intergenic Non-Protein Coding RNA 662	NA	[179]
128	*LINC00673*	Long Intergenic Non-Protein Coding RNA 673	NA	[180]
129	*LINC00963*	Long Intergenic Non-Protein Coding RNA 963	NA	[181]
130	*LINC01014*	Long Intergenic Non-Protein Coding RNA 1014	EGFR-PI3K-AKT-mTOR signalling pathway	[18]
131	*LINC01232*	Long Intergenic Non-Protein Coding RNA 1232	NA	[182]
132	*LINC01433*	Long Intergenic Non-Protein Coding RNA 1433	NA	[183]
133	*LINC01535*	Long Intergenic Non-Protein Coding RNA 1535	JAK/STAT3 signaling pathway	[21]
134	*LINC02042*	Long Intergenic Non-Protein Coding RNA 2042	c-Myc signaling pathway	[184]
135	*LINP1*	LncRNA In Non-Homologous End Joining Pathway 1	NA	[185]
136	*Lnc-ABCA12-3*	ATP Binding Cassette Subfamily A Member 12-3	NA	[186]
137	*Lnc-MCEI*	Also known as Ribosomal Protein S10 Pseudogene 7 *(RPS10P7)*	NA	[187]
138	*Lnc TUG1*	Taurine Up-Regulated 1	Wnt/β-catenin signaling pathway	[188]
139	*LOC100133669*	Also known as LY6E Divergent Transcript *(LY6E-DT)*	NA	[189]
140	*LOC440173*		NA	[190]
141	*LOC441178*		NA	[191]
142	*MACC1-AS1 and FOXD2-AS1*	MACC1 Antisense RNA 1 and Forkhead Box D2 Adjacent Opposite Strand RNA 1	NA	[61]
143	*MAFG-AS1 or MAFG-DT*	MAF BZIP Transcription Factor G Divergent Transcript	NA	[192]
144	*MIAT*	Myocardial Infarction Associated Transcript	NA	[193]
145	*MIR205HG*	MicroRNA 205 Host Gene	NA	[194]
146	*NLIPMT*	Novel lncRNA inhibiting proliferation and metastasis	NA	[195]
147	*PSMA3-AS1*	Proteasome 20S Subunit Alpha 3 Antisense RNA 1	NA	[196]
148	*PTCSC1*	Papillary Thyroid Carcinoma Susceptibility Candidate 1	Akt signaling pathway	[20]
149	*SNHG22*	Small nucleolar RNA host gene 22	NA	[197]
150	*SNHG12*	Small nucleolar RNA host gene 12	NA	[198]
151	*TTTY15*	Testis-Specific Transcript, Y-Linked 15	JAK2 signaling pathway	[199]
152	*TUSC7*	Tumor Suppressor Candidate 7	NA	[200]
153	*uc061hsf.1*		p53 signaling pathway	[201]
154	*UPK1A-AS1*	Uroplakin 1A Antisense RNA 1	NA	[202]

155	*WDFY3-AS2*	WD Repeat And FYVE Domain Containing 3 Antisense RNA 2	NA	[203]
156	*ZEB2-AS1*	Zinc Finger E-Box Binding Homeobox 2 Antisense RNA 1	NA	[204]
157	*ZFPM2-AS1*	Zinc Finger Protein, FOG Family Member 2 Antisense RNA 1	NA	[205]
158	*BAALC-AS1*	Brain And Acute Leukemia Cytoplasmic Antisense RNA 1	NA	[206]
159	*BCAR4*	Breast Cancer Anti-Estrogen Resistance 4	p53/p21 signaling pathway	[207]
160	*CASC15*	Cancer Susceptibility 15	NA	[208]
161	*DGCR5*	DiGeorge Syndrome Critical Region Gene 5	NA	[209]
162	*DIO3OS*	Iodothyronine Deiodinase 3 Opposite Strand Upstream RNA	NA	[210]
163	*FAM83A-AS1*	Family With Sequence Similarity 83 Member A Antisense RNA 1	NA	[211]
164	*GAPLINC*	Gastric Adenocarcinoma Associated, Positive CD44 Regulator, Long Intergenic Non-Coding RNA	NA	[212]
165	*GASL1 or GASAL1*	Growth Arrest Associated LncRNA 1	Wnt3a/β-catenin signaling pathway	[16]
166	*HCP5*	HLA Complex P5	PI3K/AKT/mTOR signaling pathway	[19]
167	*KCNQ1 overlapping transcript 1*	Potassium Voltage-Gated Channel Subfamily Q Member 1 overlapping transcript 1	NA	[213]
168	*LINC00239*	Long Intergenic Non-Protein Coding RNA 239	NA	[214]
169	*LINC00261*	Long Intergenic Non-Protein Coding RNA 261	NA	[215]
170	*LINC00467*	Long Intergenic Non-Protein Coding RNA 467	NA	[216]
171	*LINC00491*	Long Intergenic Non-Protein Coding RNA 491	NA	[217]
172	*LINC00551*	Long Intergenic Non-Protein Coding RNA 551	NA	[218]
173	*LINC00941*	Long Intergenic Non-Protein Coding RNA 941	NA	[219]
174	*LINC00958*	Long Intergenic Non-Protein Coding RNA 958	NA	[220]
175	*LINC01305*	Long Intergenic Non-Protein Coding RNA 1305	NA	[221]
176	*LINCIN*		NA	[222]
177	*LINC-PINT*	Long Intergenic Non-Protein Coding RNA, P53 Induced Transcript	NA	[223]
178	*LOC101928477*	Uncharacterized *LOC101928477*	NA	[224]
179	*LOC146880 or ARHGAP27P1*	Rho GTPase Activating Protein 27 Pseudogene 1	MAPK signaling pathway	[23]
180	*NEAT1*	Long Noncoding RNA Nuclear Paraspeckle Assembly Transcript 1	MDM2 signaling pathway	[225]
181	*SNHG7*	Long non-coding RNA small nucleolar RNA host gene 7	NA	[226]

182	*LOXL1-AS1*	Lysyl Oxidase Like 1 Antisense RNA 1	NA	[227]
183	*MTX2-6*	Metaxin 2-6	NA	[228]
184	*MY or VPS9D1-AS1*	VPS9 Domain Containing 1 Antisense RNA 1	NA	[229]
185	*SNHG5*	Small Nucleolar RNA Host Gene 5	NA	[230]
186	*SOX4*	SRY-Box Transcription Factor 4	NA	[231]
187	*THAP9-AS1*	THAP Domain Containing 9 Antisense RNA 1	NA	[232]
188	*TMEM161B-AS1*	Transmembrane Protein 161B Divergent Transcript	NA	[233]
189	*VESTAR*	Vascular Endothelial Growth Factor C mRNA stability-associated long noncoding RNA	NA	[234]
190	*LINC00324*	Long Intergenic Non-Protein Coding RNA 324	MAPK signaling pathway	[24]
